# Optimization of Whole Tumor Cell Vaccines by Interaction with Phagocytic Receptors

**DOI:** 10.3390/vaccines9080904

**Published:** 2021-08-14

**Authors:** Mladen Korbelik

**Affiliations:** Department of Integrative Oncology, BC Cancer, Vancouver, BC V5Z 1L3, Canada; mkorbelik@bccrc.ca; Tel.: +1-604-675-8084

**Keywords:** cancer immunotherapy, therapeutic vaccine, tumor cell vaccine, photodynamic therapy-generated vaccine, phagocytic receptors, mouse SCCVII tumor

## Abstract

The principal event in the function of whole-cell cancer vaccines is the ingestion of vaccine-delivered tumor antigen-containing material, which is performed by the patient’s antigen-presenting cells (APCs) through the employment of their phagocytic receptors. The goal of the present study was to identify the phagocytic receptors critical for the therapeutic efficacy of whole-cell cancer vaccines. The model of photodynamic therapy (PDT)-generated vaccines based on mouse SCCVII tumors was utilized, with in vitro expanded SCCVII cells treated by PDT serving as the vaccine material used for treating mice bearing established SCCVII tumors. The therapeutic impact, monitored as delayed progression of vaccinated tumors, was almost completely eliminated when antibodies specifically blocking the activity of LOX-1 scavenger receptor were administered to mice 30 min before vaccination. Similar, but much less pronounced, impacts were found with antibodies neutralizing the activity of CR3/CR4 receptors recognizing complement-opsonized vaccine cells, and with those blocking activating Fcγ receptors that recognized IgG antibody-based opsonins. A strikingly contrary action, a greatly enhanced tumor control by the vaccine, was found by blocking immune inhibitory receptor, FcγRIIB. The reported findings establish, therefore, an attractive strategy that can be effectively exploited for potent therapeutic enhancement of PDT-generated (and probably other) whole-cell tumor vaccines.

## 1. Introduction

Recent years have seen impressive advances in the development of therapeutic tumor/cancer vaccines, and there are currently several hundred such vaccines in development and undergoing clinical trials [1]. It is now firmly established that the therapeutic cancer vaccines represent a reliable and effective form of tumor immunotherapy capable of establishing an enduring anti-tumor immunity. They provide means for active immunization enabling tumor patients to build up their immune system to hold in check and specifically kill tumor cells in systemic fashion, destroying metastatic deposits and preventing tumor recurrence [2]. The principal types of these vaccines are tumor cell vaccines, dendritic cell vaccines, peptide vaccines, oncoviral or bacterial vector vaccines, and nucleic acid vaccines [3].

Whole-cell tumor vaccines have been the focus of our research for almost two decades. Autologous vaccines of this type are prepared from surgically removed tumor tissues of patients receiving treatment. Their advantage is targeting optimally at once multiple unknown and known tumor antigens and thus avoiding the immune escape caused by tumor antigen loss [4]. Moreover, these vaccines are conditioned to express individualized pertinent and even unique antigens in a patient-specific manner provided in patient-matched MHC for recognizing tumor peptides [4,5]. To render tumor vaccine cells non-proliferative and non-tumorigenic while retaining, for a while, metabolic activity, they are usually exposed immediately before vaccination to a lethal X-ray dose that was shown not to compromise their immunogenicity [6]. Such polyvalent vaccines are conditioned to raise responses to a variety of both MHC class I and class II epitopes destined to stimulate a wide range of cellular and humoral anti-tumor immune responses. Importantly, surgically removed tissue can be directly used for the vaccine, thus obviating the procedure of cellular culture establishment and associated risks, delays, and restrictions [7]. Non-cancerous cells from tumor tissue are generally found to be non-immunogenic; moreover, they are phagocytized differently from cancer cells. No evidence of complications due to the induction of immunity against normal cells was found in clinical trials with autologous vaccines [4]. A potential disadvantage with these vaccines is the requirement of an adequate amount of harvested tumor tissue.

Since the vaccines made of intact tumor cells regularly fail to elicit an effective immune response, a variety of interventions have been introduced to amplify their immunizing potential [4]. One such extremely effective intervention was shown to be exposing the vaccine tumor cells ex vivo to rapid tumor ablation treatments, such as photodynamic therapy (PDT) or photothermal therapy (PTT) [5,8]. These treatments are highly proficient in pronouncedly elevating the immunogenicity of targeted tumor cells by triggering oxidative or thermal stress signaling that (i) induces their immunogenic cell death (ICD), and (ii) magnifies their antigenic fingerprint through the expression of cryptic and novel tumor antigens due to altered (unconventional) translation activity [9,10].

One of the principal features of the mechanism of anti-tumor immune response development instigated by tumor cell vaccines is the mobilization of efferocytosis (cell disposal) pathways instrumental to the presentation of tumor antigens delivered in the vaccine material [5]. The potency of these vaccines is critically dependent on the engagement of key elements of efferocytosis, including phagocytic cell types and the activity of their phagocytic receptors [11]. The aim of the present study was to identify phagocytic receptors whose participation is essential for the therapeutic efficacy of tumor cell vaccines.

## 2. Materials and Methods

The therapeutic tumor vaccine model used in most of our studies is based on mouse squamous cell carcinoma SCCVII, a recognized immunotherapy model for head and neck cancer [12]. For PDT-generated vaccines, in vitro expanded SCCVII cells were incubated with photosensitizer ce6 (Frontier Scientific, Logan, UT, USA) at 1 μg/mL for 30 min with subsequently exposure to 665 ± 10 nm light (1 J/cm^2^) followed by a 16 h incubation in growth conditions [13]. The cells were then exposed to a lethal X-ray dose and immediately thereafter used as a vaccine by injecting (2 × 10^7^ cells/mouse) peritumorally into SCCVI tumor-bearing mice. The vaccine effects were assessed by monitoring changes in tumor size. As noticed in our earlier studies [7], the ranges of response within the same treatment group usually vary greatly from completely inhibited to uninhibited tumor growth progression. This diversity results in huge standard deviation bars that prevent meaningful standard student t-test-like statistical analysis. Hence, expressing the results as the percentage of tumors that were significantly growth-inhibited is more informative. The tumors smaller than the means minus two-fold standard deviation of the control group qualified as growth-inhibited. As a part of standard vaccine protocol, the mice received cyclophosphamide (50 mg/kg, i.p) 4 days after vaccination in order to block immunoregulatory T cells. Antibodies blocking the activity of mouse phagocytic receptors were injected i.p. (30 μg/mouse) 30 min before vaccine administration. They included anti-LOX1 and anti-FcγRI/CD64 (both goat IgG, from R&D Systems, Minneapolis, MN, USA), anti-macrophage class A scavenger receptor-A (anti-SR-A) goat polyclonal sc-20444 from Santa Cruz Biotechnology Inc. (Dallas, TX, USA), anti-CD18 (clone 2E6 from hybridoma HB-226), and antibody recognizing Fcγ receptor epitope common to FcγRIIB and FcγRIII (rat IgG2b clone 2.4G2 from HB-197 hybridoma).

Each treatment group consisted of six mice, and there were at least two repeat independent experiments. A log-rank test was used for statistical analysis of tumor growth inhibition results, with the threshold for statistical significance set at 5% (*p* < 0.05). With this test, calculations are performed for each data time point comparing estimates of hazard functions of the two groups (vaccine alone versus vaccine plus blocking antibody) in the same way as for Kaplan–Meier survival curves.

## 3. Results and Discussion

**Figure 1:**
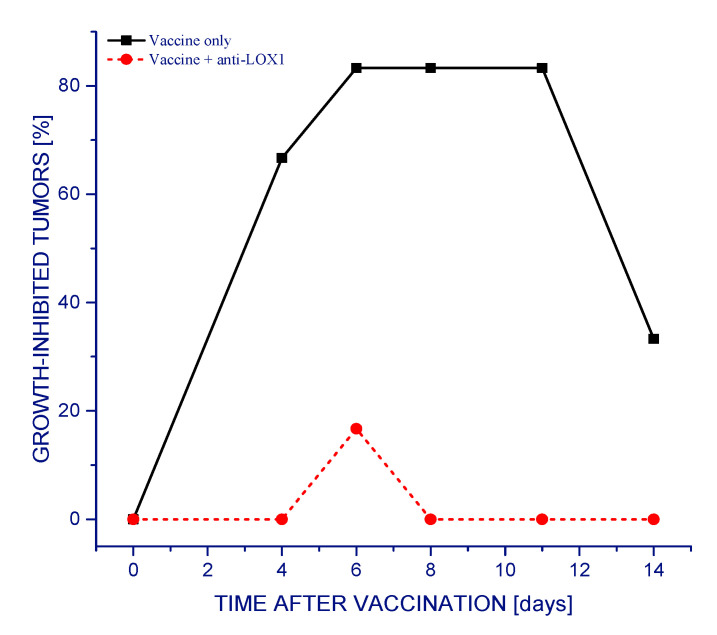
The effect of blocking LOX-1 receptor on the therapeutic efficiency of PDT-generated vaccines. Mice bearing SCCVII tumors received a peritumoral injection of a vaccine consisting of SCCVII cells treated in vitro by PDT and processed as described in Materials and Methods. LOX-1 neutralizing antibodies were injected into mice (30 μg/mouse i.p.) 30 min before vaccine administration. The response to therapy was assessed by tumor size measurement, and is presented as the percentage of growth-inhibited tumors (smaller than the means minus two-fold standard deviation of unvaccinated control group). The response to the PDT vaccine plus anti-LOX1 (circles) is statistically different from vaccine only (squares) group (*p* < 0.05; *n* = 6).

Professional antigen-presenting cells (APCs) from the patient are responsible for processing the captured antigen material from the administered tumor cell vaccine, and presenting antigenic peptides on their surface for the recognition by T lymphocytes in tumor-draining lymph nodes [4]. This is initiated by ingesting vaccine cells by APCs (particularly macrophages and dendritic cells) through the engagement of their phagocytic receptors [11]. The present study investigated the importance of the involvement of particular receptors of this type for the overall therapeutic efficacy of the vaccine. The results in Figure 1 demonstrate that almost all SCCVII tumors exhibited reduced growth rates following a single vaccine injection. Dendritic cells and other APCs were shown to be attracted to the vaccine injection site, and, with peritumoral administration, this facilitates the accumulation of activated T cells in tumor-draining lymph nodes [5]. This therapeutic effect of the vaccine was dramatically eliminated with antibodies injected into mice 30 min before vaccination that block the interaction of LOX-1 receptor with its key ligand phosphatydilserine (PS) [14] (Figure 1). In the long-term follow-up, one third of the vaccine alone-treated mice remained tumor-free at 90 days post therapy (qualifying them as cured), while mice treated with vaccine plus anti-LOX-1 exhibited progressively growing tumors that needed to be euthanized within 30 days after therapy (not shown).

**Figure 2:**
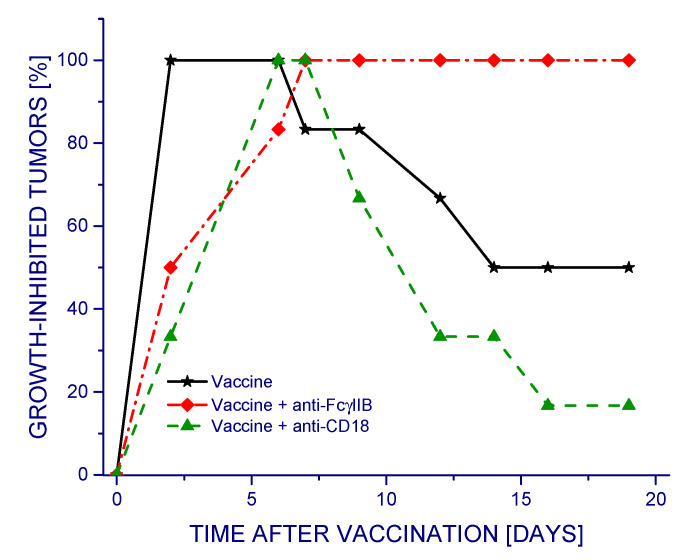
The effect of blocking complement CR3/CR4 receptors (CD18) or immune inhibitory receptor FcγRIIB on the therapeutic efficacy of PDT-generated vaccines. Mice bearing SCCVII tumors received a peritumoral injection of vaccine cells prepared as described in Figure 1. Antibodies blocking specific phagocytic receptors were injected into mice (30 μg/mouse i.p.) 30 min before vaccine administration. The response to therapy is presented as explained in Figure 1. The response to both groups, PDT vaccine plus anti-CD18 (triangles), and PDT plus anti-FcγRIIB (rombs), is statistically different from the vaccine only (stars) group (*p* < 0.05; *n* = 6).

Different outcomes were obtained when testing in the same way the antibodies 2.4G2 and 2E4 that specifically prevent the engagement of phagocyte-specific immune inhibitory receptor FcγRIIB [15] or β2 integrin-based complement receptors CR3 and CR4 [11], respectively (Figure 2). In this case, the treatment with vaccine alone proved highly effective in reducing tumor growth rates during the first 10 days post vaccination and then continued to be effective in approximately one half of the tumors. Neutralizing the activity of receptors CR3 and CR4 by the 2E4 antibody (anti-CD18) produced a negative impact, as demonstrated by the reduced therapeutic efficacy of the vaccine. This effect was not evident with data presented as average tumor volumes for each group with standard deviations (Supplementary Figure S1). Remarkably, the 2.4G2 antibody strongly augmented the efficacy of the vaccine, rendering it 100% effective in inhibiting tumor growth throughout the observation period. Although this antibody recognizes both activating FcγRIII and inhibitory FcγRIIB receptors, the former receptor is low affinity and biologically of minor relevance in mice, which makes 2.4G2 effective specifically for neutralizing murine FcγRIIB [16].

The results of testing additional phagocytic receptor-blocking antibodies and comparing their effects to the finding presented in Figure 1 and Figure 2 are summarized in Table 1. It can be seen that the greatest positive therapeutic impact was attained by blocking FcγRIIB. All other tested antibodies produced negative impacts, with the exception of SR-A neutralization, which had no significant effect. The vaccine efficacy was largely eliminated by antibodies blocking LOX-1, while this effect was somewhat less pronounced with anti-CD18 and anti-FCγRI. Treatment with isotype (non-specific) controls for all the above antibodies had no detectable impact on vaccine response.

Our earlier work has established that the potency of whole-cell tumor vaccines generated by PDT is optimized when time is allowed for the expression of PDT-induced apoptotic death changes before the vaccine material is injected [7]. This work suggested that one such key apoptotic signal is cell surface-expressed PS, which is in a complete agreement with the current finding that the LOX-1 phagocytic receptor specified for binding PS is totally indispensable for the therapeutic effect of the investigated tumor cell vaccines. Supportive, but less essential roles were revealed by other phagocytic receptors, including CR3 and CR4 receptors recognizing complement-opsonized vaccine cells and activating Fcγ receptors recognizing IgG antibody-based opsonins [11].

Blocking FcγRIIB will prevent its inhibiting the initiation of an activating signaling cascade by multiple activating FcγRs [15]. Activating FcγRs include the high affinity FcγRI and low affinity receptor family comprising FcγRIIA, FcγRIIC, FcγRIIIA, and FcγRIIIB in humans, and FcγRIII and FcγRIV in mice. The present report establishes that blocking the inhibitory receptor FcγRIIB can be effectively exploited for potent therapeutic enhancement of PDT-generated (and probably other) whole tumor cell vaccines. This highlights a novel strategy for optimization of whole-cell vaccines by focusing on phagocyte-controlling mechanisms based on immune inhibitory receptors that have been identified in increasing numbers on phagocytes [17]. These receptors control distinct mechanisms for regulating specific phagocytic functions, allowing the fine-tuning of patients’ phagocytes for the potentiation of the therapeutic performance of whole-cell vaccines.

## Figures and Tables

**Table 1:** The impact of blocking various phagocytic receptors on the efficacy of PDT cancer vaccines.

Phagocytic Receptor Blocked	Impact on PDT Cancer Vaccine Efficacy
FcγRIIB (2.4G2 Ab)	Greatly enhanced ^#^
FcγRI (anti-CD64 Ab)	Reduced *
SR-A	No significant effect
LOX-1	Completely obstructed ^&^
CR3/CR4	Reduced *

Blocking antibodies were, in all cases, used at 30 μg/mouse i.p. and administered 30 min before vaccination. ^#^ All tumors growth-inhibited; * Tumor growth retardation less pronounced than with vaccine alone group; ^&^ No significant tumor growth retardation.

## Data Availability

All pertinent previously unpublished data is contained within the article.

## Supplementary Materials

The following are available online at https://www.mdpi.com/article/10.3390/vaccines9080904/s1, Figure S1: The effect of blocking complement CR3/CR4 receptors (CD18) or immune inhibitory receptor FcγRIIB on the therapeutic efficacy of PDT-generated vaccines.
