# Supplementary material for: Optimization of Whole Tumor Cell Vaccines by Interaction with Phagocytic Receptors
Source: Vaccines (Basel). 2021 Aug 14;9(8):904. doi: 10.3390/vaccines9080904 (PMC8402491; doi:10.3390/vaccines9080904)
Supplement: Supplementary file 1 [file vaccines-09-00904-s001.zip › vaccines-1344266-supplementary.pdf]

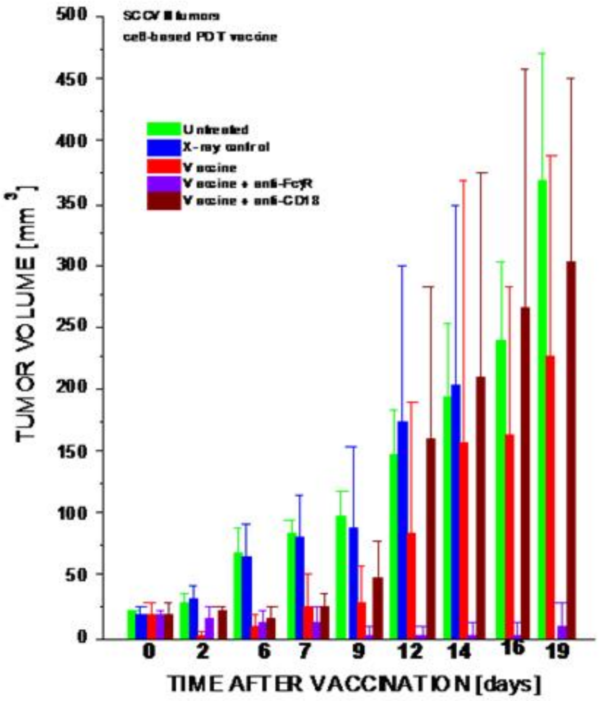

Supplementary Figure S1. The effect of blocking complement CR3/CR4 receptors (CD18) or immune inhibitory receptor FcγRIIB on the therapeutic efficacy of PDT-generated vaccines. Mice-bearing SCCVII tumors received peritumoral injection of vaccine cells prepared from SCCVII cells as described for Fig. 1. Antibodies blocking specific phagocytic receptors were injected into mice (30 μg/mouse i.p.) 30 min before vaccine administration. The response to therapy was assessed by tumor size measurement, and is presented as means for tumor volume plus standard deviations. An extra control group consisting of SCCVII cells exposed to x-ray treatment only is also included.
